# 2023 at *PLOS Biology*

**DOI:** 10.1371/journal.pbio.3002474

**Published:** 2023-12-20

**Authors:** The PLOS Biology Staff Editors

**Affiliations:** Public Library of Science, San Francisco, California, United States of America and Cambridge, United Kingdom

## Abstract

2023 saw many important advances in the life sciences. In this editorial, we highlight research from across the breadth of *PLOS Biology*’s scope.

2023 is drawing to a close, and it has been a big year for *PLOS Biology*. We have celebrated our 20^th^ anniversary by publishing articles throughout the year that look back at landmark *PLOS Biology* papers, take stock of whole research fields or take a deep dive into the future of science publishing (they can all be found on our 20^th^ anniversary collection page). We have also sadly said goodbye to our neuroscience section manager Kris Dickson and our microbiology/immunology editor Paula Jauregui, welcoming Christian Schnell and Melissa Vazquez Hernandez to the team in those roles. They have hit the ground running and are already actively engaging with their communities—do reach out to them, or indeed to any of our staff editors if you would like to talk about your work. We will soon have details on our website of how to book video calls during “office hours” with the team, but for now an email is a good way to request a chat.

The past year has also been big for advances in the life sciences, seeing the first synthetic human embryo [1], the first sequence of the human Y chromosome [2], and the first successful functional transplant of a cryopreserved kidney in rats [3]. More worryingly, 2023 was also the year that we passed the safe limit on the sixth planetary boundary [4] and experienced record high temperatures around the world. The life sciences will have an important role in addressing the challenges facing our planet, which we chose to highlight in our collections on Biology for planetary sustainability and Engineering plants for a changing climate.

Within our pages, we have also published some excellent research in 2023. To round off the year, we highlight a varied selection of articles that underscores the breadth of our scope, and which we hope will make for enjoyable holiday reading.

Starting with molecular biology, “Functional unknomics: Systematic screening of conserved genes of unknown function” by Rocha et al [5] was a remarkable study. Here, the authors developed a publicly available “unknome” database that ranks proteins on the basis of how little is known about them. Using a genetic screen in *Drosophila*, they focus on this unknome and show that these genes contribute to a wide range of fundamental biological processes. These findings demonstrate the importance of neglected genes in biological research and provide a resource to investigate unexplored regions of the proteome.

Among our microbiology content, one of our favorites was “Defining the minimal components of the influenza A virus replication machinery via an in vitro reconstitution system” from the Fodor lab [6] for its apparent simplicity and elegance. The influenza virus genome is composed of eight RNA segments and in this study the authors establish the first completely *in vitro* system for the full transcription and replication of the influenza virus ribonucleoproteins, defining the essential set of viral and host factors necessary for these processes. Using this system, the authors provide new insights into the role of different components in influenza replication and set the stage to study replication of other negative-sense RNA viruses, as well as to, for example, screen for inhibitors of viral replication.

In immunology, a highlight was “The activity of the aryl hydrocarbon receptor in T cells tunes the gut microenvironment to sustain autoimmunity and neuroinflammation” by Merchack et al [7], which explored the role of the aryl hydrocarbon receptor (AHR) in T cells in multiple sclerosis. Here, the absence of AHR reduced the T cell pool while increasing metabolite production in the gut microbiome, pointing to a novel function of AHR as a regulator of the gut environment. This study links gut microbiome activity with autoimmune responses in the central nervous system, providing new molecular targets for the potential treatment of autoimmune diseases.

Among our human physiology content, “Day-night and seasonal variation of human gene expression across tissues” by Wucher et al [8] provided a substantial resource for the chronobiology field. The authors analyzed transcriptomic data from GTEx and combined this with information about time and season of death, detailing gene expression changes elicited by the day-night cycle and by seasonal changes across a wide variety of human tissues. These findings help lay a foundation for understanding the fascinating effects of circadian and circannual influences on human biology.

Within our neuroscience section, “Optogenetic and chemogenetic approaches reveal differences in neuronal circuits that mediate initiation and maintenance of social interaction” by Rojek-Sito et al [9] stood out. In it, the authors studied how two different aspects of dynamic social interactions, the initiation and maintenance of social contact, are regulated in rat brains. They found that initiating and maintaining social interactions are controlled by distinct multi-circuit networks. These findings highlight the complexity of the regulation of social behavior and enhance our understanding of specific aspects of social behavior.

Looking at plant biology, “Multiple light signaling pathways control solar tracking in sunflowers” by Brooks et al [10] was a highlight. In it, the authors analyzed heliotropism (sun-tracking) in sunflowers and, by comparing gene expression patterns with other plants, found that this phenomenon is not a specialized form of phototropism, as had been previously thought, but rather a different type of directional light response that involves multiple light signaling pathways, which they are now investigating.

A paper from our ecology section with beautiful images was “High concentrations of floating neustonic life in the plastic-rich North Pacific Garbage Patch” by Chong, Spencer et al [11]. The authors used samples from the North Pacific Garbage Patch to show that this major ocean gyre contains high levels of floating neustonic species, as well as the well-known anthropogenic plastic debris. This sort of data will help inform strategies to protect and conserve the oceans and complements our recent collection of articles on “Ocean solutions for a sustainable, healthy and inclusive future”.

Finally, in our meta-research section, a highlight was “The manifold costs of being a non-native English speaker in science” by Amano et al [12]. The authors used questionnaire responses from more than 900 scientists to quantify the diverse costs to non-native English-speaking researchers of the fact that much of global science is conducted and communicated in this one language. As well as putting numbers to a problem that we all know exists, and showing that it impacts multiple aspects of scientific life, we liked the fact that the study also offers potential solutions.

Our heartfelt thanks to all of the authors, reviewers and Academic Editors who have contributed to *PLOS Biology* in 2023 and who, in doing so, have supported the PLOS mission for open science. We hope that 2024 brings more discoveries in the life sciences that will change the world for the better.
